# Author Correction: Mutant p53 drives clonal hematopoiesis through modulating epigenetic pathway

**DOI:** 10.1038/s41467-020-17555-0

**Published:** 2020-07-28

**Authors:** Sisi Chen, Qiang Wang, Hao Yu, Maegan L. Capitano, Sasidhar Vemula, Sarah C. Nabinger, Rui Gao, Chonghua Yao, Michihiro Kobayashi, Zhuangzhuang Geng, Aidan Fahey, Danielle Henley, Stephen Z. Liu, Sergio Barajas, Wenjie Cai, Eric R. Wolf, Baskar Ramdas, Zhigang Cai, Hongyu Gao, Na Luo, Yang Sun, Terrence N. Wong, Daniel C. Link, Yunlong Liu, H. Scott Boswell, Lindsey D. Mayo, Gang Huang, Reuben Kapur, Mervin C. Yoder, Hal E. Broxmeyer, Zhonghua Gao, Yan Liu

**Affiliations:** 1 Department of Biochemistry and Molecular Biology, Indiana University, Indianapolis, IN 46202 USA; 2 Department of Pediatrics, Herman B Wells Center for Pediatric Research, Indiana University, Indianapolis, IN 46202 USA; 3 Department of Biochemistry and Molecular Biology, the Cancer Institute, College of Medicine, Pennsylvania State University, Hershey, PA 17033 USA; 4 Department of Microbiology and Immunology, Indiana University, Indianapolis, IN 46202 USA; 5 Shanghai Municipal Hospital of Traditional Chinese Medicine, Shanghai University of Traditional Chinese Medicine, Shanghai, China; 6 Department of Medical Genetics, Indiana University, Indianapolis, IN 46202 USA; 7 Department of Ophthalmology, Indiana University, Indianapolis, IN 46202 USA; 8 Siteman Cancer Center, Washington University, St. Louis, MO 63110 USA; 9 Department of Medicine, Indiana University, Indianapolis, IN 46202 USA; 10 Division of Pathology and Experimental Hematology and Cancer Biology, Cincinnati Children’s Hospital Medical Center, Cincinnati, OH USA

**Keywords:** Stem cells, Haematopoietic stem cells

Correction to: *Nature Communications* 10.1038/s41467-019-13542-2, published online 11 December 2019.

The original version of this article omitted references to previous work in ‘Bondar, T. & Medzhitov, R. p53-mediated hematopoietic stem and progenitor cell competition. *Cell Stem Cell* **6**, 309–322 (2010)’ and ‘Marusyk, A., Porter, C. C., Zaberezhnyy, V. & DeGregori, J. Irradiation selects for p53-deficient hematopoietic progenitors. *PLoS Biol.* **8**, e1000324 (2010)’. These have been added as references 23 and 24 in the Introduction, the original sentence ‘We discovered that mutant p53 enhances the repopulating potential of HSPCs^22^’ has been amended to ‘We discovered that mutant p53 enhances the repopulating potential of HSPCs^22^, similar to what has been reported previously^23,24^.’ This has been corrected in the PDF and HTML versions of the article.

