# Supplementary material for: Mutant p53 drives clonal hematopoiesis through modulating epigenetic pathway
Source: Nat Commun. 2019 Dec 11;10:5649. doi: 10.1038/s41467-019-13542-2 (PMC6906427; doi:10.1038/s41467-019-13542-2)
Supplement: Supplementary file 2 — Supplementary Information [file 41467_2019_13542_MOESM2_ESM.pdf]

Supplementary Information

Mutant p53 Drives Clonal Hematopoiesis through Modulating Epigenetic Pathway

Chen et al.

Supplementary Figure 1

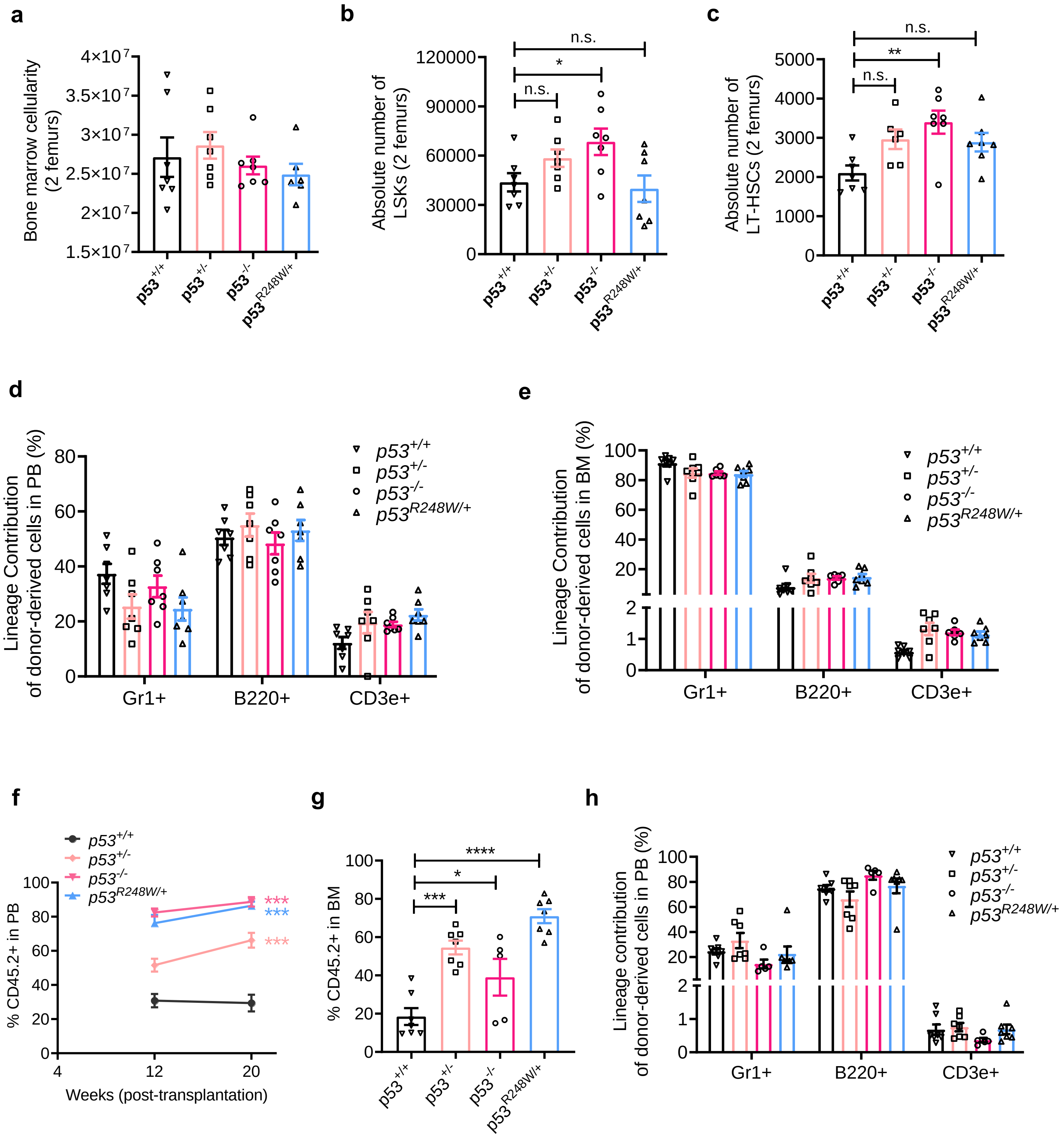

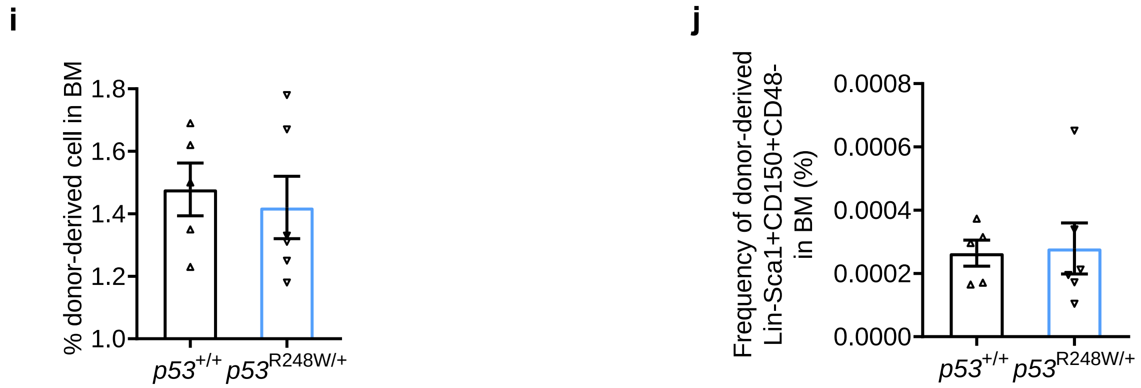

Supplementary Figure 1. *p53*$^{R248W/+}$ HSPCs show enhanced repopulating potential. (**a**) Bone marrow (BM) cell numbers from *p53*$^{+/+}$, *p53*$^{+/-}$, *p53*$^{-/-}$, and *p53*$^{R248W/+}$ mice (8 to 12 weeks old). n=6-7 mice per group. (**b**) The absolute number of LSKs in two femurs of *p53*$^{+/+}$, *p53*$^{+/-}$, *p53*$^{-/-}$, and *p53*$^{R248W/+}$ mice. n=6-7 mice per group. (**c**) The absolute number of LT-HSCs (CD48^{-}CD150^{+}LSKs) in two femurs of *p53*$^{+/+}$, *p53*$^{+/-}$, *p53*$^{-/-}$, and *p53*$^{R248W/+}$ mice. n=6-7 mice per group. (**d**) Lineage contribution of donor-derived cells in the peripheral blood (PB) of the primary recipient mice at 20 weeks following HSC transplantation was determined by flow cytometry analysis. n =7 mice per group. (**e**) Lineage contribution of donor-derived cells in the BM of the primary recipient mice at 20 weeks following HSC transplantation was determined by flow cytometry analysis. n =7 mice per group. (**f**) Percentage of donor-derived cells in the PB of recipient mice following secondary transplantation. n=7 mice per group. (**g**) Percentage of donor-derived cells in the BM of recipient mice at 20-week after secondary transplantation. n=7 mice per group. (**h**) Lineage contribution of donor-derived cells in the PB of recipient mice at 20 weeks following secondary transplantation was determined by flow cytometry analysis. n =7 mice per group. (**i**) 1 x 10^{7} BM cells (CD45.2^{+}) from *p53*$^{+/+}$ and *p53*$^{R248W/+}$ mice were transplanted into lethally irradiated recipient mice (CD45.1^{+}). At 18 hours after transplantation, the percentage of donor-derived cells in the BM of recipient mice was determined by flow cytometry. n=5-6 mice per group. (**j**) The frequency of donor-derived Lin^{-}Sca1^{+}CD48^{-}CD150^{+} cells in the BM of recipient mice 18 hours after transplantation was determined by flow cytometry. n=5-6 mice per group. Data are represented as mean ± SEM. p-values were calculated using one-way ANOVA with Dunnett's multiple comparisons test in **a**, **b**, **c**, and **g**, two-way ANOVA with Dunnett's multiple comparison test in **d, e, f** and **h,** unpaired t-test with Welch's correction in **i** and **j**; *P<0.05, **P<0.01, ***P<0.001, ****P<0.0001. Source data are provided as a Source Data file.

Supplementary Figure 2

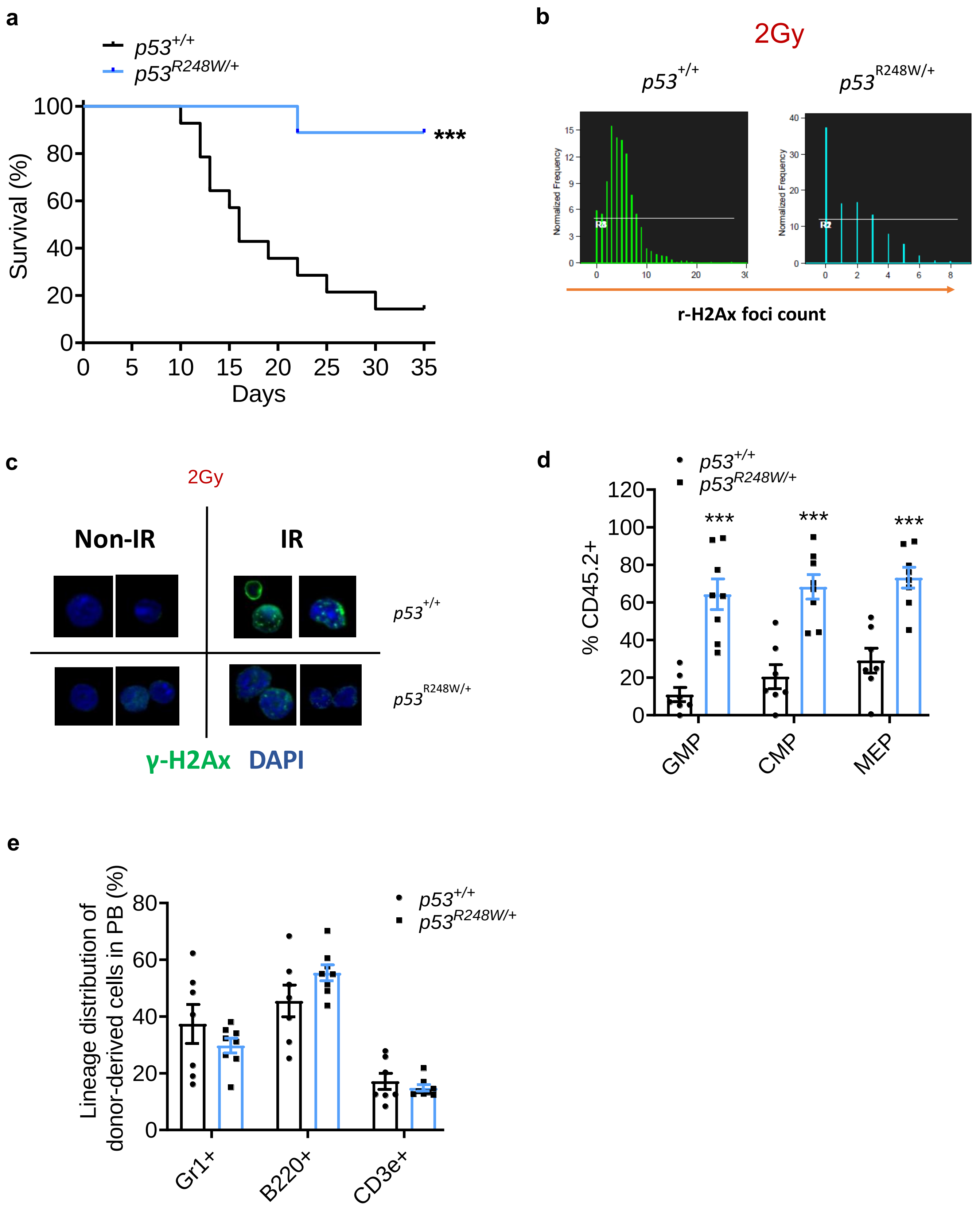

Supplementary Figure 2. *p53*$^{R248W/+}$ HSPCs are not sensitive to irradiation.
(**a**) Kaplan-Meier survival curve of *p53*$^{+/+}$ and *p53*$^{R248W/+}$ mice after 9Gy total body irradiation (TBI). *p53*$^{+/+}$ n = 14 mice, and *p53*$^{R248W/+}$ n = 9 mice. (**b**) Fluorescence intensity of γ-H2AX in LT-HSCs at 2 hours after 2Gy irradiation was determined by ImageStream flow cytometry analysis. (**c**) γ-H2AX foci generation in HSCs following irradiation. LT-HSCs from *p53*$^{+/+}$ and *p53*$^{R248W/+}$ mice were immunostained for γ-H2AX at 2 hours after 2Gy irradiation. LT-HSCs were stained with DAPI to identify the nuclei. (**d**) The frequency of donor-derived MEPs, CMPs, and GMPs in the BM of primary recipient mice 16 weeks following transplantation was determined by flow cytometry analysis. n = 7-8 mice per group. (**e**) Lineage contribution of donor-derived cells in the PB of the primary recipient mice 16 weeks following transplantation was determined by flow cytometry analysis. n = 7-8 mice per group. Data are represented as mean ± SEM. p-values were calculated using log-rank (Mantel–Cox) test in **a**, unpaired t-test with Welch's correction in **d**, two-way ANOVA with Dunnett's multiple comparison test in **e**; ***P<0.001. Source data are provided as a Source Data file.

Supplementary Figure 3

a

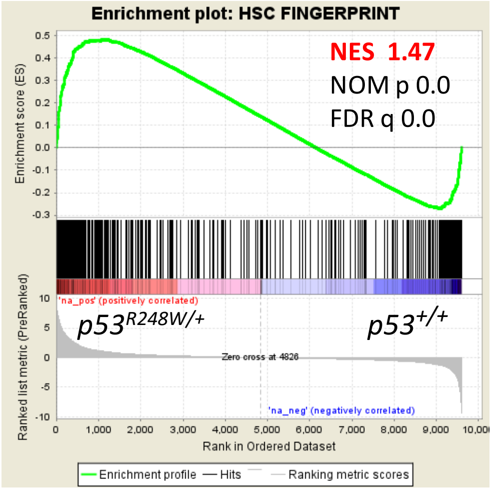

b

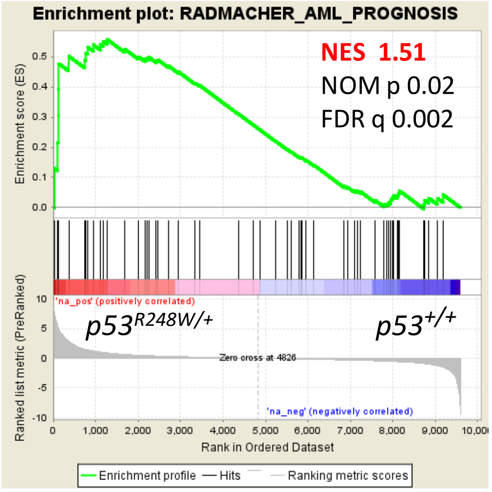

c

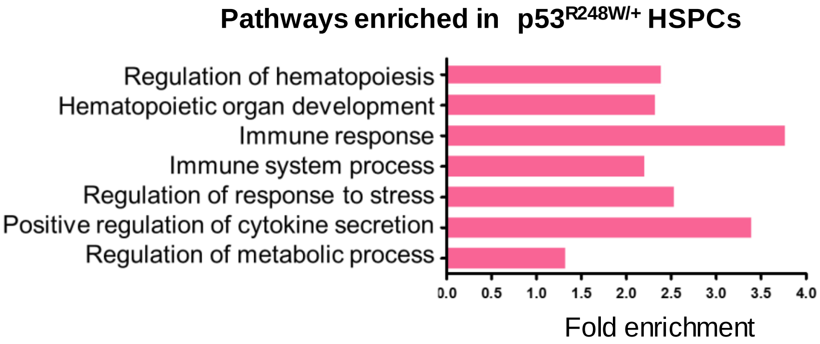

d

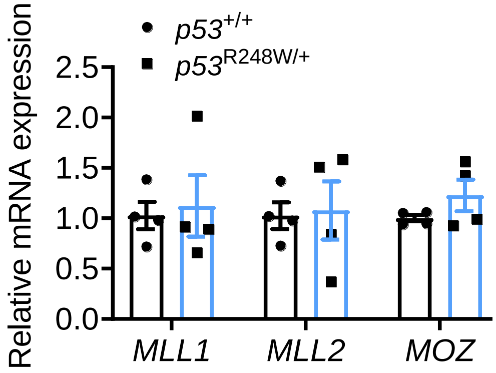

e

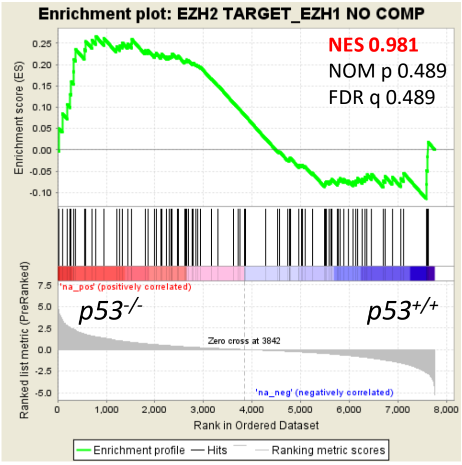

Supplementary Figure 3. *p53*$^{R248W/+}$ HSPCs show enriched HSC signatures.
(**a**) Gene Set Enrichment Analysis (GSEA) identified hematopoietic stem cell (HSC) related gene sets are enriched in p53 mutant HSPCs compared to wild-type HSPCs in Molecular Signature Database (MsigDB). (**b**) GSEA analysis show enrichment of AML signatures in p53 mutant HSPCs compared to wild-type HSPCs. (**c**) DAVID pathway analysis of genes upregulated in p53 mutant HSPCs compared to wild-type HSPCs. (**d**) Quantitative RT-PCR analysis of the mRNA levels of *MLL1, MLL2,* and *MOZ* in HSCs. n=4 biological replicates. (**e**) GSEA analysis of EZH2 target genes in *p53*$^{+/+}$ and *p53*$^{-/-}$ LSKs. Data are represented as mean ± SEM. p-values were calculated using GSEA software in **a**, **b**, and **e**, GO analysis in **c**, unpaired t-test with Welch's correction in **d**. Source data are provided as a Source Data file.

Supplementary Figure 4

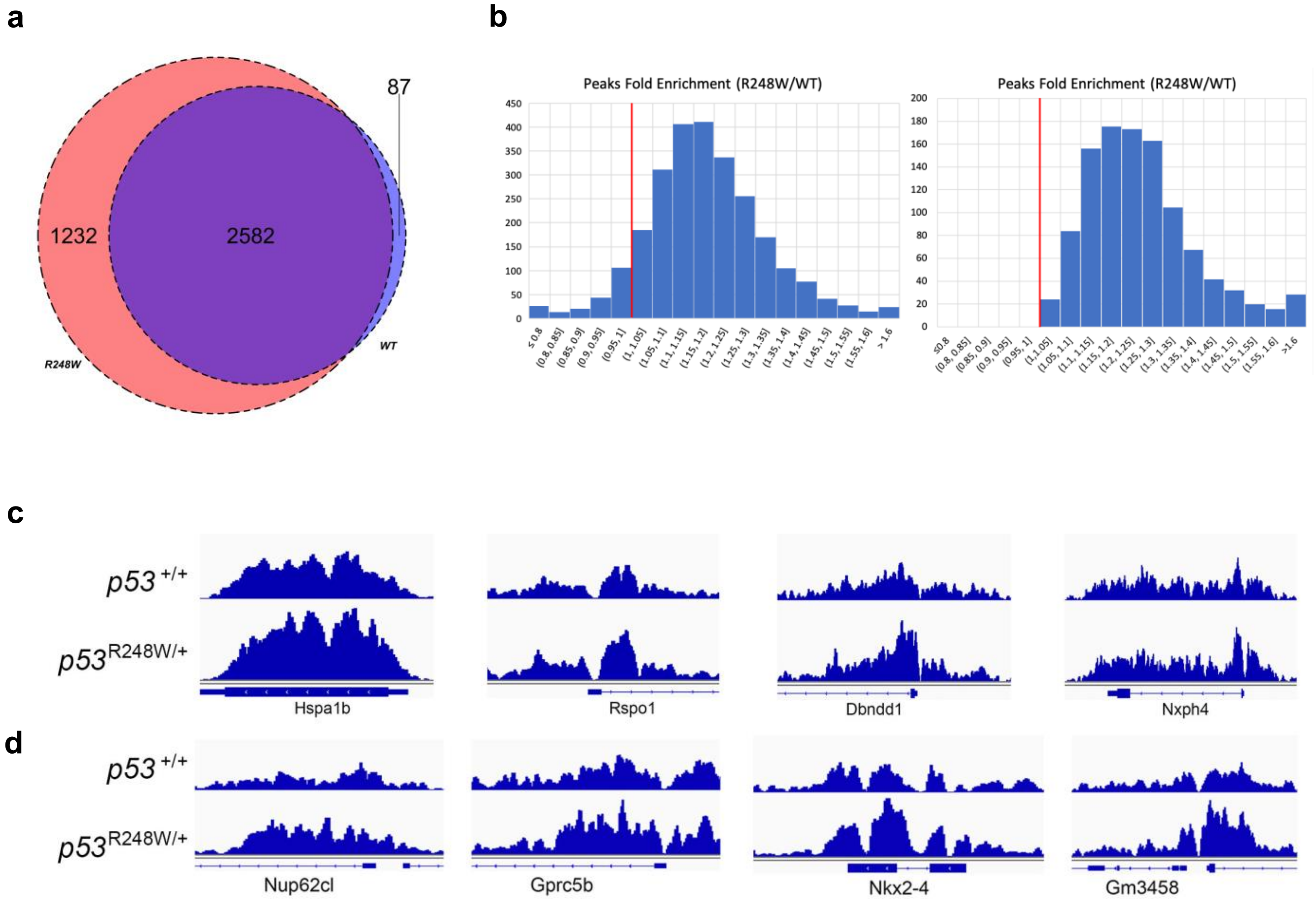

Supplementary Figure 4. *p53*$^{R248W/+}$ enhances genome wide H3K27me3 in HSPCs. (**a**) Venn diagram of H3K27me3 ChIP-seq peaks in p53$^{R248W/+}$ HSPCs compared to p53$^{+/+}$ HSPCs. (**b**) Histogram showing the distribution of relative enrichment (peaks fold enrichment) of H3K27me3 peaks in p53$^{R248W/+}$ HSPCs compared to that of the p53$^{+/+}$ HSPCs. On the left are peaks shared by both cell types and on the right are those peaks detected in mutant cells but fell below the threshold in WT cells. Note that the majority of peaks have a relative enrichment >1 (as indicated by the red line). (**c**), (**d**) Genome browser views of H3K27me3 intensities on selected loci that are from two group of cells as shown in b. Source data are provided as a Source Data file.

Supplementary Figure 5

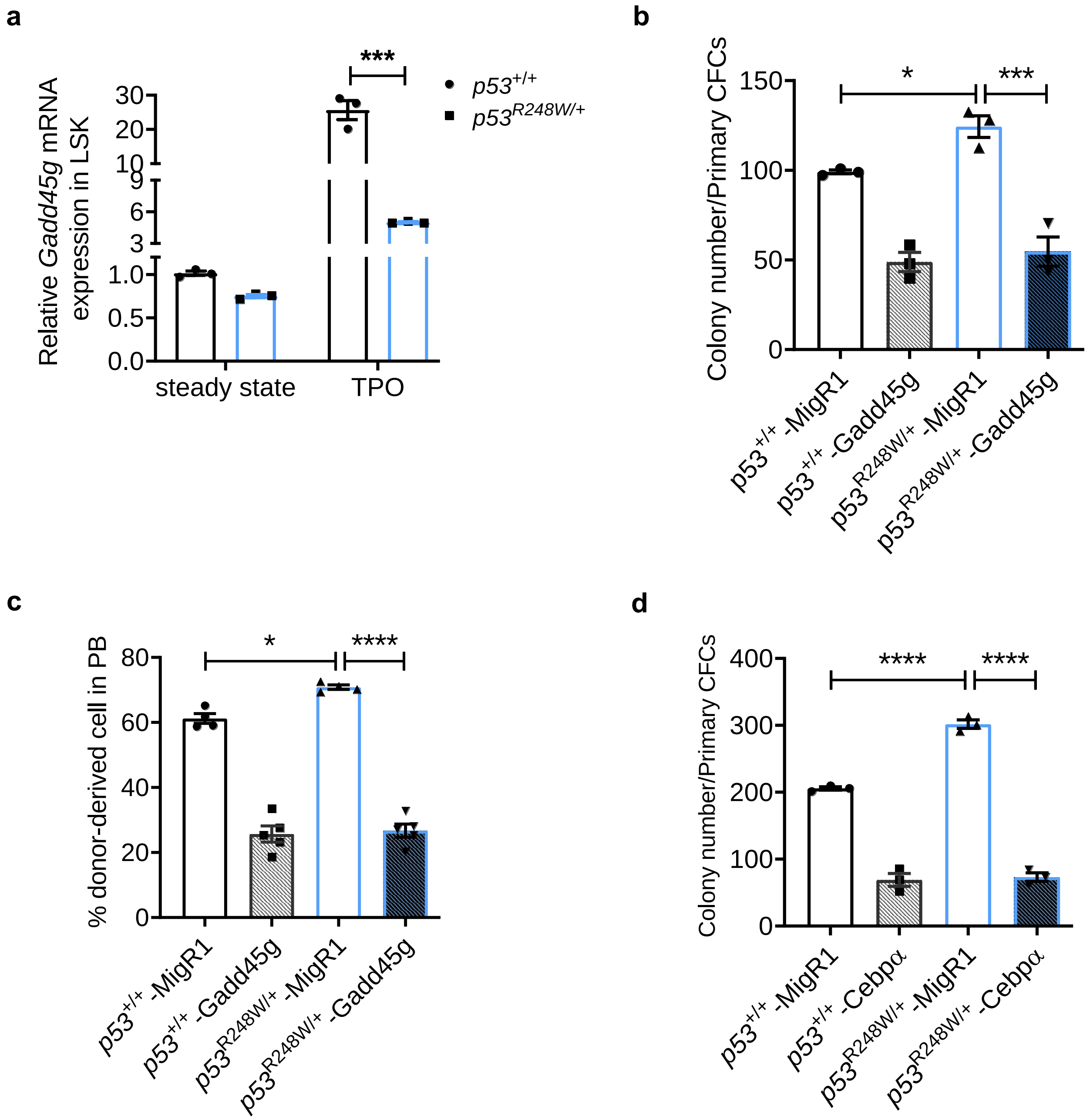

Supplementary Figure 5. Overexpression of *Gadd45g* decreases the repopulating potential of p53 mutant HSPCs. (**a**) Quantitative RT-PCR analysis of mRNA levels of *Gadd45g* in HSPCs with or without TPO stimulation. n=3 biological replicates. (**b**) Ectopic *Gadd45g* expression decreases the colony formation of p53 mutant BM cells. n = 3 independent experiments performed in duplicate. (**c**) Ectopic *Gadd45g* expression decreases repopulating potential of p53 mutant BM cells at 8 weeks following competitive transplantation. n = 4-5 mice per group. (**d**) Ectopic *Cebpα* expression decreases the colony formation of p53 mutant BM cells. n = 3 independent experiments performed in triplicate. Data are represented as mean ± SEM. p-values were calculated using two-way ANOVA with Bonferroni's multiple comparisons test in **a**, and one-way ANOVA with

Turkey's multiple comparison test in **b, c** and **d**; *$P<0.05$, ***$P<0.001$, ****$P<0.0001$. Source data are provided as a Source Data file.

Supplementary Figure 6

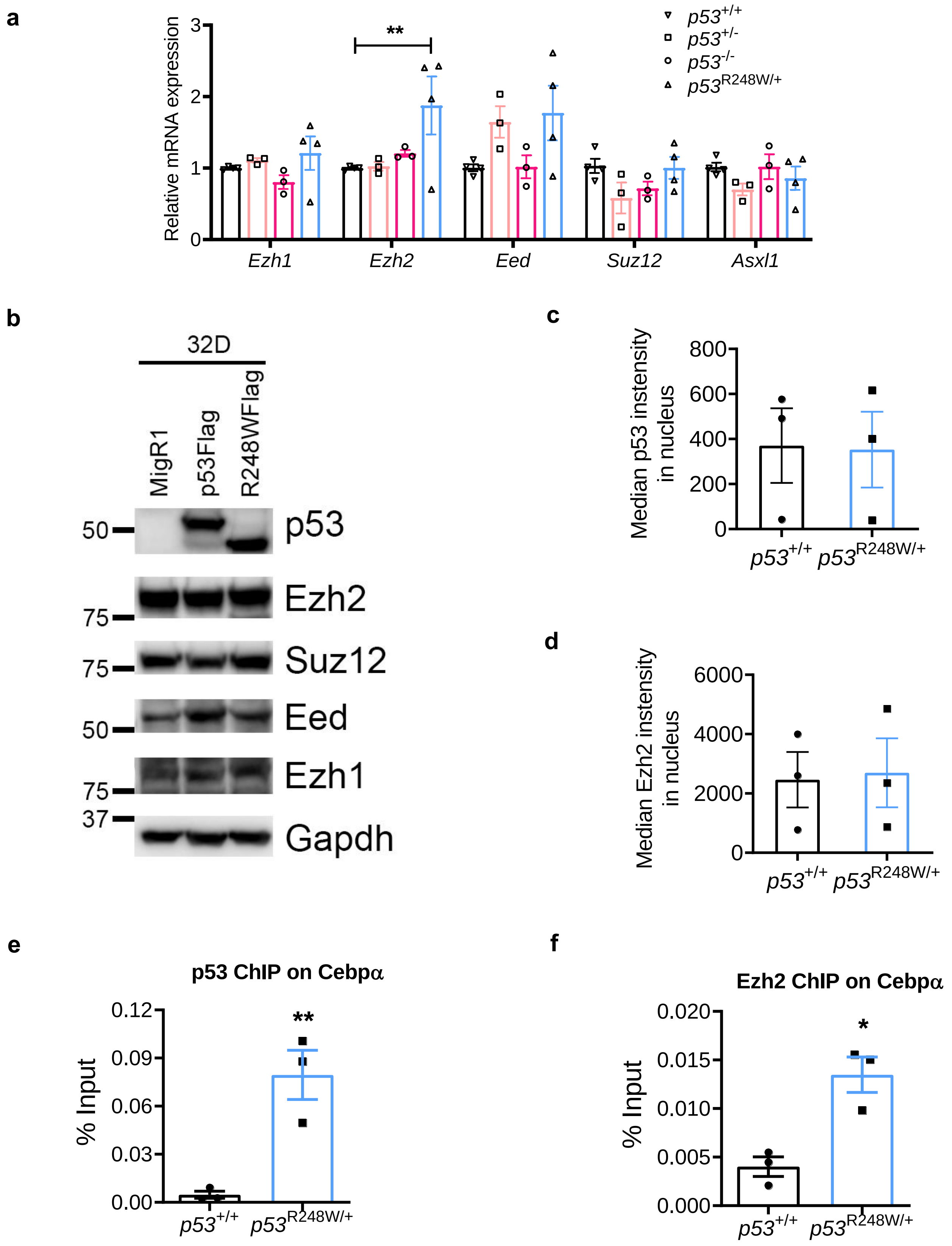

Supplementary Figure 6. Mutant p53 interacts with EZH2 in HSPCs. (**a**) The expression of the components of the PRC2 complex, including *Ezh1*, *Ezh2*, *Eed*, and *Suz12*, in HSPCs was determined by quantitative RT-PCR analysis. n=3-4 biological replicates. (**b**) Ectopic expression of wild-type (WT) or mutant p53 did not affect the protein levels of the PRC2 components in 32D cells. (**c**) Median fluorescent intensity (MFI) of p53 in the nucleus of *p53*$^{+/+}$ and *p53*$^{R248W/+}$ HSPCs (CD150$^+$LSKs). n=3 biological replicates. (**d**) MFI of Ezh2 in the nucleus of *p53*$^{+/+}$ and *p53*$^{R248W/+}$ HSPCs (CD150$^+$LSKs). n=3 biological replicates. (**e**) p53 enrichment on *Cebpα* in *p53*$^{+/+}$ and *p53*$^{R248W/+}$ HSPCs were examined by p53-ChIP assays. n= 3 independent experiments. (**f**) EZH2 enrichment on *Cebpα* in *p53*$^{+/+}$ and *p53*$^{R248W/+}$ HSPCs were examined by EZH2-ChIP assays. n= 3 independent experiments. Data are represented as mean ± SEM. p-values were calculated using two-way ANOVA with Dunnett's multiple comparisons test in **a**, paired t-test in **c**, **d**, **e** and **f**; *P<0.05, **P<0.01. Source data are provided as a Source Data file.

Supplementary Figure 7

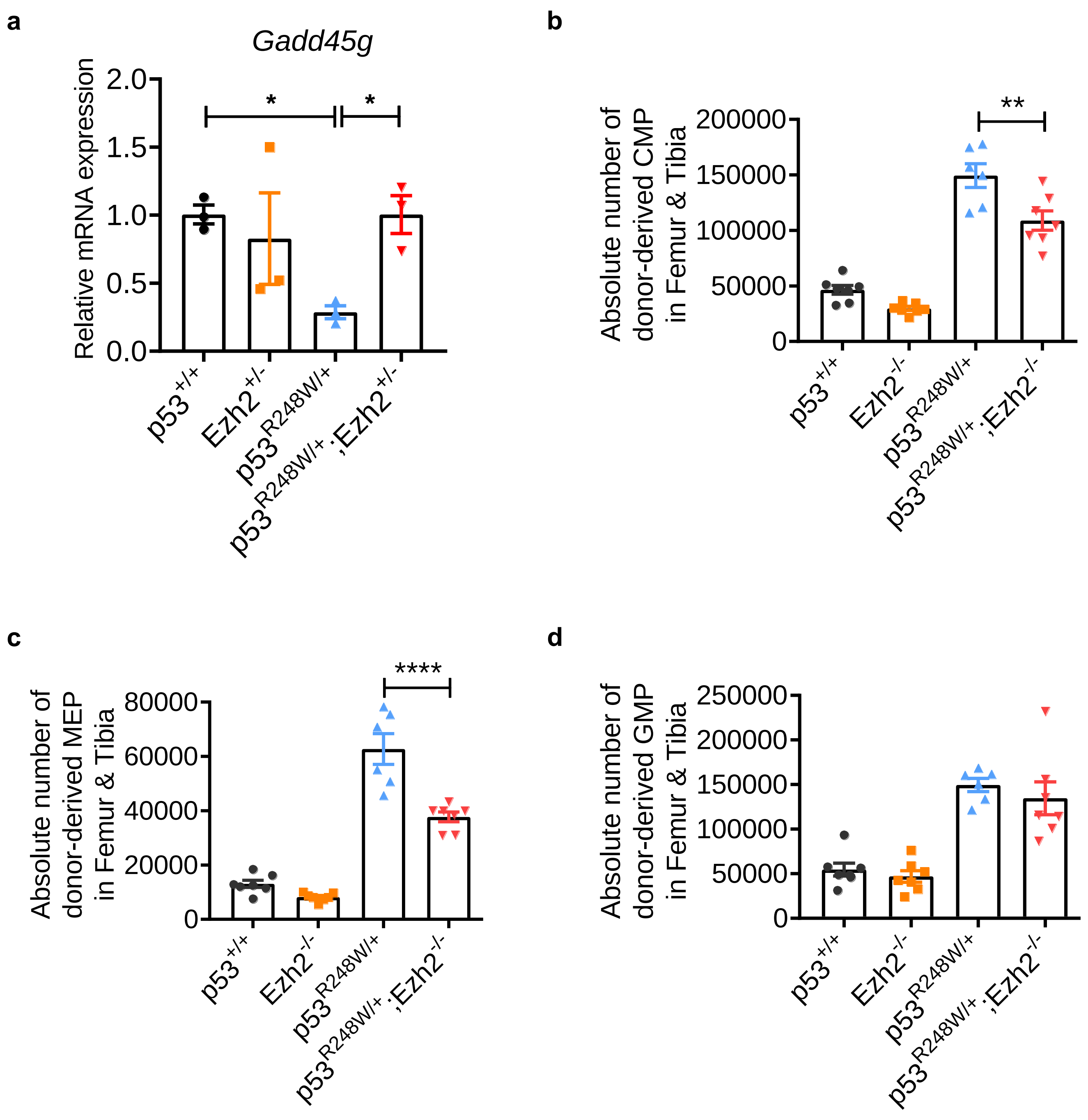

Supplementary Figure 7. Loss of EZH2 decreases the repopulating potential of p53 mutant HSPCs. (**a**) *Gadd45g* expression in $p53^{+/+}$, $Ezh2^{+/-}$, $p53^{R248W/+}$ and $p53^{R248W/+}$ $Ezh2^{+/-}$ HSPCs. n=3 biological replicates. (**b**) The absolute number of donor-derived CMPs in one femur and one tibia of recipient mice 20 weeks following pI:pC treatment. n =6-7 mice per group. (**c**) The absolute number of donor-derived MEPs in one femur and one tibia of recipient mice 20 weeks following pI:pC treatment. n =6-7 mice per group. (**d**) The absolute number of donor-derived GMPs in one femur and one tibia of

recipient mice 20 weeks following pI:pC treatment. n =6-7 mice per group. Data are represented as mean ± SEM. p-values were calculated using one-way ANOVA with Turkey's multiple comparisons test; $^{**}P<0.01$, $^{***}P<0.001$, $^{****}P<0.0001$. Source data are provided as a Source Data file.

Supplementary Figure 8

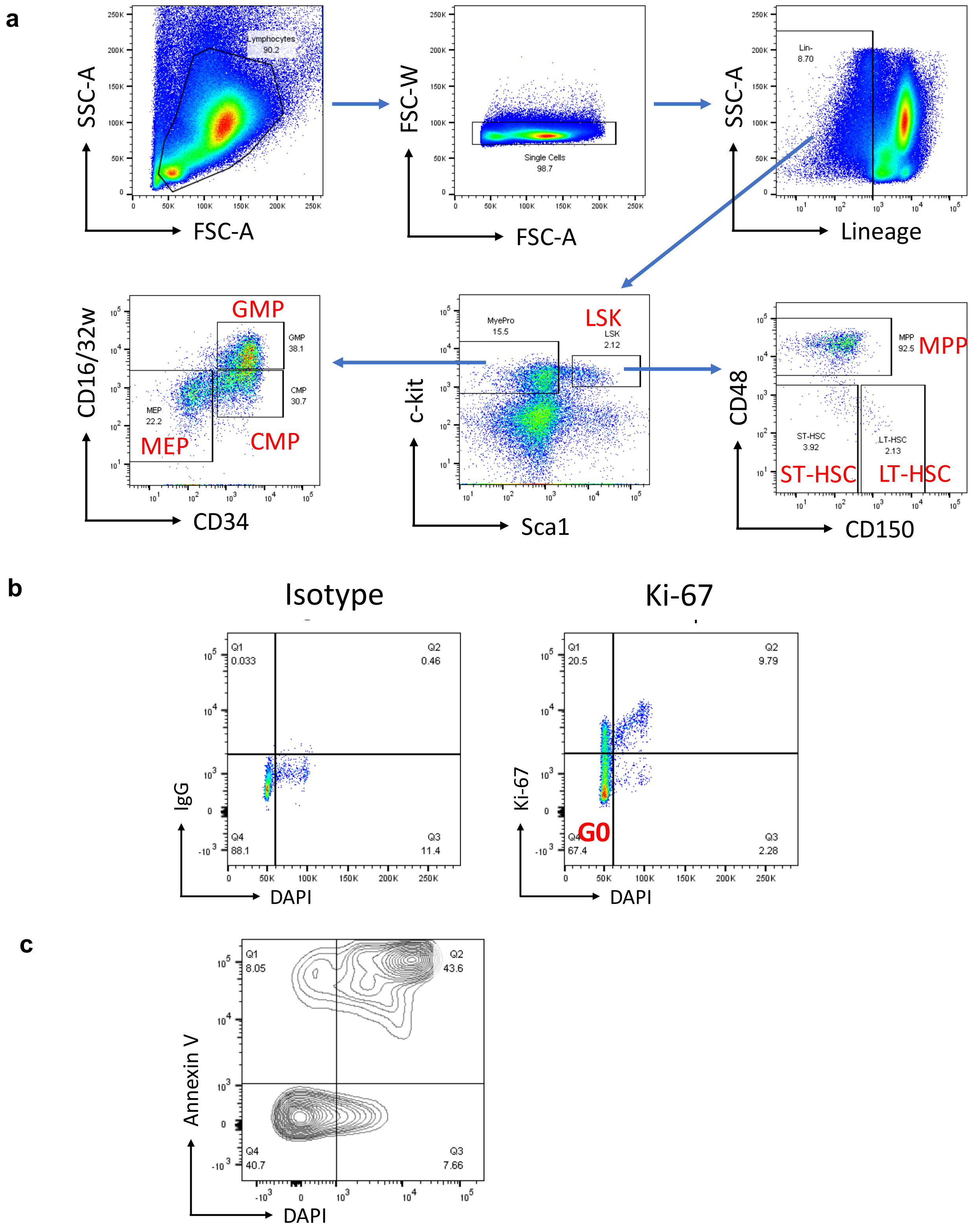

Supplementary Figure 8. Flow cytometry analysis of HSPCs. (**a**) Gating strategy for HSPC flow cytometry analysis. (**b**) Flow cytometry analysis of ki-67 staining in LSK cells. (**c**) Flow cytometry analysis of apoptosis by Annexin V and DAPI staining in LSK cells.

Table 1. List of antibodies used in the study

CATALOG #	COMPANY	ANTIBODY	CLONE	DILUTION
		**FACS antibodies or dyes**		
109806	Biolegend	FITC anti-mouse CD45.2 Antibody	104	$4x10^6$ cells/1ul
109814	Biolegend	APC anti-mouse CD45.2 Antibody	104	$4x10^6$ cells/1ul
110708	Biolegend	PE anti-mouse CD45.1 Antibody	A20	$4x10^6$ cells/1ul
405208	Biolegend	APC Cy7 Streptavidin	-	$4x10^6$ cells/1ul
115922	Biolegend	PerCP/Cy5.5 anti-mouse CD150 (SLAM) Antibody	D7	$4x10^6$ cells/1ul
105812	BioLegend	APC anti-mouse CD117 (c-Kit) Antibody	2B8	$4x10^6$ cells/1ul
105814	Biolegend	PE/Cy7 anti-mouse CD117 (c-Kit) Antibody	2B8	$4x10^6$ cells/1ul
105826	Biolegend	APC Cy7 - anti mouse CD117 Ckit	2B8	$4x10^6$ cells/1ul
12117183	eBioscience	PE-anti mouse CD117	2B8	$4x10^6$ cells/1ul
103412	Biolegend	APC anti-mouse CD48 Antibody	HM48-1	$4x10^6$ cells/1ul
110721	Biolegend	Pacific Blue™ anti-mouse CD45.1 Antibody	A20	$4x10^6$ cells/1ul
11034185	eBioscience	FITC- Anti-mouse CD34	RAM34	$2x10^6$ cells/1ul
101318	Biolegend	PE/Cy7 anti-mouse CD16/32 Antibody	93	$4x10^6$ cells/1ul
108428	Biolegend	PerCP/Cyanine5.5 anti-mouse Ly-6G/Ly-6C (G	RB6-8C5	$4x10^6$ cells/1ul
100204	BioLegend	FITC anti-mouse CD3 Antibody [Clone: 17A2] 5	17A2	$4x10^6$ cells/1ul
100320	Biolegend	PE/Cy7 anti-mouse CD3ε Antibody (100 µg)	17A2	$4x10^6$ cells/1ul
108120	Biolegend	Pacific Blue™ anti-mouse Ly-6A/E (Sca-1) Ant	D7	$4x10^6$ cells/1ul
420403	Biolegend	7-AAD Viability Staining Solution (200 tests)		1:50
133307	Biolegend	Biotin anti-mouse/human CD45R/B220	RA3-6132	$4x10^6$ cells/1ul
133307	Biolegend	Biotin anti-mouse Lyt-6G/Ly-6C (Gr-1)	RB6-8C5	$4x10^6$ cells/1ul
133307	Biolegend	Biotin anti-mouse CD3ε	145-2C11	$4x10^6$ cells/1ul
133307	Biolegend	Biotin anti-mouse TER-119/ Erythroid cells	TER-119	$4x10^6$ cells/1ul
556027	Biosciences	PE Mouse Anti-Ki-67 Set	B56	$2x10^6$ cells/1ul
D9542-10MG	Sigma	DAPI		1ug/ml
		**Western Blot antibodies**		
5246S	Cell Signalling	EZH2	AC22	'1:1000
sc-85283	Santa Cruz Biotechnology	ASXL1	SC-293204	'1:200
C36B11	Cell Signaling	H3K27me3	C36B11	'1:1000
ab176115	abcom	EZH1	AB64850	'1:1000
3737S	Cell Signaling	SUZ12	D39F6	'1:1000
09-774	Millipore	EED		'1:1000
sc-6243	Santa Cruz Biotechnology	p53	DO-1	'1:200
2524S	Cell Signaling	p53	1C12	'1:200
07-448	Millipore	H3K27me1		'1:1000
MA5-15738	Thermo Fisher Scientific	Gapdh	GA1R	'1:5000
ab1791	abcom	H3		'1:5000
